# Increased expression of the oxidative pentose phosphate pathway and gluconeogenesis in anaerobically growing xylose-utilizing *Saccharomyces cerevisiae*

**DOI:** 10.1186/1475-2859-8-49

**Published:** 2009-09-24

**Authors:** David Runquist, Bärbel Hahn-Hägerdal, Maurizio Bettiga

**Affiliations:** 1 Department of Applied Microbiology, Lund University, PO Box 124, SE-221 00 Lund, Sweden

## Abstract

**Background:**

Fermentation of xylose to ethanol has been achieved in *S. cerevisiae *by genetic engineering. Xylose utilization is however slow compared to glucose, and during anaerobic conditions addition of glucose has been necessary for cellular growth. In the current study, the xylose-utilizing strain TMB 3415 was employed to investigate differences between anaerobic utilization of glucose and xylose. This strain carried a xylose reductase (*XYL1 *K270R) engineered for increased NADH utilization and was capable of sustained anaerobic growth on xylose as sole carbon source. Metabolic and transcriptional characterization could thus for the first time be performed without addition of a co-substrate or oxygen.

**Results:**

Analysis of metabolic fluxes showed that although the specific ethanol productivity was an order of magnitude lower on xylose than on glucose, product yields were similar for the two substrates. In addition, transcription analysis identified clear regulatory differences between glucose and xylose. Respiro-fermentative metabolism on glucose during aerobic conditions caused repression of cellular respiration, while metabolism on xylose under the same conditions was fully respiratory. During anaerobic conditions, xylose repressed respiratory pathways, although notably more weakly than glucose. It was also observed that anaerobic xylose growth caused up-regulation of the oxidative pentose phosphate pathway and gluconeogenesis, which may be driven by an increased demand for NADPH during anaerobic xylose catabolism.

**Conclusion:**

Co-factor imbalance in the initial twp steps of xylose utilization may reduce ethanol productivity by increasing the need for NADP+ reduction and consequently increase reverse flux in glycolysis.

## Introduction

Production of fuel ethanol has increased several fold during the last decade due to increasing oil prices and environmental concerns [1]. The vast majority of this production comes from fermentation of agricultural products, primarily sugar cane and corn, by baker's yeast *S. cerevisiae*. Lignocellulose biomass from forest and agricultural residues is an alternative to sucrose (sugar cane) and starch (corn) based ethanol production [2,3]. Next to glucose, the main component of lignocellulose is xylose, and the use of this substrate by *S. cerevisiae *has been enabled through expression of heterologous enzymes [4-6]. Xylose utilizing *S. cerevisiae *strains have been constructed by expressing a reduction/oxidation pathway involving xylose reductase (XR) and xylitol dehydrogenase (XDH) [7,8] or a xylose isomerase (XI) pathway [9-11].

Successive cycles of metabolic engineering have improved xylose utilization in recombinant *S. cerevisiae *[12,13]. Compared to glucose however the ethanol productivity from xylose is still low. Poor xylose utilization has been ascribed to potentially rate-controlling metabolic steps including: low substrate affinity of the recombinant enzymes [8]; cofactor imbalance in the XR-XDH reactions [7,14]; low xylose transport capacity [15,16]; and failure to recognize xylose as a fermentable carbon source [17,18]. Among several experimental approaches, glucose and xylose metabolism have been investigated by transcriptional analysis to identify rate-controlling processes in xylose metabolism [17,19-22]. Growing cells are needed to establish (pseudo) steady-state conditions for transcription analysis and determination of metabolic fluxes [23,24]. The analysis of xylose utilizing strains has thus been hampered by poor anaerobic growth on xylose. Transcription analysis has consequently been conducted under aerobic conditions [17,19,20,22] and/or with addition of glucose as a co-substrate [21]. Transcriptional characterization of anaerobic xylose metabolism has however remained elusive, regardless of the importance of this particular condition in a production setting.

For *S. cerevisiae *expressing the oxidoreductive xylose assimilating pathway, a recent accomplishment has been alteration of the cofactor specificity of XR through site directed mutagenesis [25-27]. By increasing the affinity of the *P. stipitis *XR for NADH, the objective has been to improve cofactor recycling in the XR-XDH coupled reactions. The current study utilized a *S. cerevisiae *strain harboring a mutated XR (K270R) with significantly improved substrate uptake rate and ethanol productivity [26]. The strain grew anaerobically on xylose as a sole carbon source which for the first time enabled quantitative metabolic flux determination and genome wide transcriptional analysis. The focus of the study was to compare metabolic fluxes during anaerobic glucose and xylose growth, and to analyze the observed differences on a transcriptional level.

## Materials and methods

### Strains and cultivation conditions

*S. cerevisiae *strains and plasmids used in this study are summarized in Table 1. *Escherichia coli *strain DH5α was used for sub-cloning and was grown on LB medium supplemented with 100 mg/L ampicillin. Defined mineral medium was used for *S. cerevisiae *cultivation and was composed of: xylose or glucose, 60 g/L; mineral salts ((NH_4_)_2_SO_4_, 5 g/L; KH_2_PO_4_, 3 g/L; MgSO_4_·7H_2_O, 0.5 g/L); buffer (potassium hydrogen phthalate, 50 mM pH 5.5); Tween 80, 0.4 g/L; ergosterol, 0.01 g/L [28]; vitamins and trace elements [29]. Identical medium was used for pre-cultures and batch fermentation in instrumented bioreactors with the exception that buffering agent was omitted in the latter case. At the start of each experiment, yeast strains were streaked from 15% (v/v) glycerol stocks and grown two days on Yeast Nitrogen Base (YNB) glucose plates. Pre-cultures were inoculated in baffled shake-flasks (10% liquid volume) at a predetermined cell density, OD_620 nm _= 0.5/0.025 (xylose/glucose), and grown for 20 hrs to yield cells in late exponential phase (OD_620 nm_~14). Cultivation of *S. cerevisiae *was performed at 30°C.

**Table 1:** *S. cerevisiae *strains and plasmids used in this study.

**Plasmids and Strains**	**Relevant Features**	**Reference**
Plasmids		
YIpOB9	*URA3 TDH3p-XYL1(K270R)-ADH1t, PGK1p-XYL2-PGK1t*	[55]
YIplac128	*LEU2*	[56]
*S. cerevisiae *strains		
TMB 3043	CEN.PK 2-1C Δ*GRE3, his3::PGK1p-XKS1-PGK1t, TAL1::PGK1p-TAL1-PGK1t, TKL1::PGK1p-TKL1-PGK1t, RKI1::PGK1p-RKI1-PGK1t, RPE1::PGK1p-RPE1-PGK1t, leu2, ura3*	[13]
TMB 3662	TMB 3043, *ura3*::YIpOB9, *leu2*	[55]
TMB 3415	TMB 3662, *leu2*::YIplac128	This work

**Figure 1:**
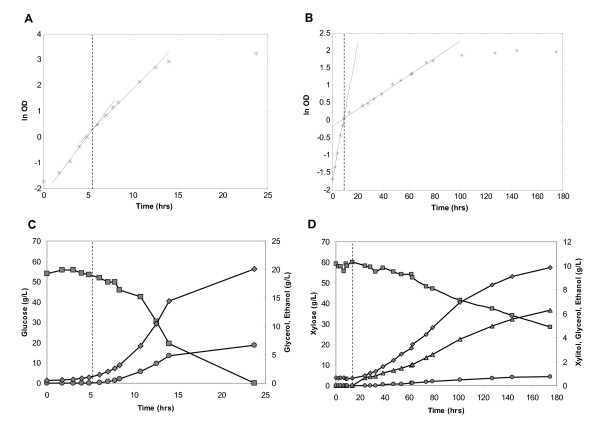
**Fermentation profiles of glucose and xylose growth**. The dashed vertical line indicates switch between aerobic and anaerobic conditions. Samples for transcription analysis were collected at OD_620 nm _~ 1 and OD_620 nm _~ 4, which corresponds ln OD ~ 0 and ln OD ~ 1.4 in the figure. **A**. Biomass production during glucose cultivation. **B**. Biomass production during xylose cultivation **C**. Substrate consumption and metabolite formation during glucose cultivation. **D**. Substrate consumption and metabolite formation during xylose cultivation. Symbols: glucose/xylose, "squares"; ethanol, "diamonds"; glycerol, "circles"; xylitol, "triangles" and biomass, "stars".

Anaerobic batch cultivation was performed in an instrumented bioreactor (Applikon Biotechnology, AC Schiedam, the Netherlands) with 1.5 L working volume and a starting OD_620 nm _of 0.2. The medium contained 60 g/L of glucose or xylose and was prepared as described above with antifoam (Dow Corning, Midland, USA) added to the reactor at a final concentration of 0.2 mL/L. Temperature was maintained at 30°C and the pH was controlled at 5.5 through addition of 3 M KOH. Cultures were grown under aerobic conditions until the cell density reached OD_620 nm _= 1.0, upon which conditions were changed to anaerobiosis for the remainder of the experiment (Figure 1). During the aerobic phase, the culture was sparged with air at a flow rate of 0.4 L/min and the stirring was set to 500 rpm. During the anaerobic phase, oxygen free conditions were maintained by nitrogen (> 99.995%) sparging at a flow rate of 0.2 L/min and the stirring rate was reduced to 200 rpm. Dissolved oxygen (DO) was monitored using a DO probe and CO_2 _production was detected online by an INNOVA 1313 fermentation monitor (LumaSense Technologies, Ballerup, Denmark). Cultures were sampled for HPLC, OD_620 nm _and dry cell weight measurements. Samples were collected for transcriptome analysis during exponential aerobic (OD_620 nm_~1.0) and anaerobic growth (OD_620 nm_~4.0). Transcriptome samples (50 mL) were collected from the bioreactor into a pre-chilled (-80°C) glass bottle. Samples were immediately centrifuged (5000*g, 2 min, 4°C) and the biomass pellet was frozen in liquid nitrogen. Experiments were performed in biological duplicate.

### Strain construction

*S. cerevisiae *strain TMB 3415 was constructed from the TMB 3043 (Table 1) parent strain [13]. This genetic background encompasses genetic changes previously identified as beneficial for xylose utilization. Genes for the non-oxidative pentose phosphate pathway [12] and xylulokinase *XKS1 *[30] have been over-expressed, and the non-specific aldose reductase gene *GRE3 *has been deleted [31]. Genes encoding *Pichia stipitis *mutated xylose reductase (*XYL1 *K270R) and xylitol dehydrogenase (*XYL2*) were integrated in TMB 3043 by transformation with YIpOB9 (Table 1) linearized with *Eco*RV, using the Lithium Acetate method [32]. The resulting strain, TMB 3662, was rendered prototrophic by integration of the linearized (*Eco*RV) vector YIplac128 (Table 1), yielding strain TMB 3415. Standard molecular biology techniques were employed [33] and Fermentas GeneJet plasmid miniprep kit (Fermentas, Vilnius, Lithuania) was used for plasmid extraction.

### Calculation of metabolic fluxes

Exponential anaerobic growth was confirmed by linear regression of the natural logarithm of cell concentration against time. Specific rates of product formation and substrate consumption were calculated in Matlab (Matlab R2007b, The MathWorks Inc., MA, USA) using Equation 1 & 2 and measured metabolite and cell concentrations. A pseudo-steady state assumption was validated by observing constant specific production- and consumption rates within 2-3 cell duplications, as well as good agreement of measured values between biological replicates.

(1)

(2)

Where: X = biomass (g/L); Met = metabolite concentration (g/L); μ = specific growth rate (h^-1^); r = specific production rate (g/h×gDW).

### Microarray analysis

Microarray analysis was performed on cell samples collected from aerobic and anaerobic batch cultivation on glucose and xylose as described above. RNA from two independent biological cultivations was analyzed. Total RNA was extracted from frozen cell pellets using a bead-beater (Biospecs products, Bartlesville, OK, USA) and Trizol reagent (Invitrogen, CA, USA). All extractions where performed on the same total amount of cells (approximately 10 mg dry weight). RNA was further purified using the RNeasy mini kit (Qiagen, Hilden, Germany). RNA quality and concentration were measured using an Agilent 2100 bioanalyzer (Agilent Technologies, Santa Clara, CA, USA) and Nanodrop ND-1000 (Thermo Fisher Scientific, Waltham, MA, USA) respectively. Total RNA was processed using the GeneChip^® ^Expression 3'-Amplification Reagents One-cycle cDNA synthesis kit (Affymetrix Inc, Santa Clara, CA, USA) to produce double-stranded cDNA. This was used as a template to generate biotin-targeted cRNA following the manufacturer's specifications. Fifteen μg of the biotin labeled cRNA was fragmented to strands between 35 and 200 bases in length, 10 μg of which was hybridised onto the GeneChip^® ^genome array overnight in the GeneChip^® ^Hybridisation oven6400 using standard procedures. The arrays were washed and then stained in a GeneChip^® ^Fluidics Station 450. Scanning was performed with the GeneChip^® ^Scanner 3000 and image analysis was performed using GeneChip^® ^Operating Software. The RMA algorithm [34] was used for normalization and scaling of the raw signal data. Student's t-test was used to identify genes with significantly (p ≤ 0.05) altered gene expression. All array data is presented as fold changes, i.e. the log2 ratio of expression signals.

### Analysis of substrate and products

Metabolite concentrations were determined by HPLC using a Waters HPLC system (Milford, MA, USA). An Aminex HPX-87H ion-exchange column (Bio-Rad, Hercules, CA, USA) and a refractive index detector (RID-6a, Shimadzu, Kyoto, Japan) were used for separation and detection, respectively. The column temperature was 45°C and 5 mM H_2_SO_4 _was used as a mobile phase at a flow rate of 0.6 mL/min. Cell dry weight was determined by filtering a known sample volume through a dried and pre-weighed 0.45-μm pore membrane (Pall Corporation, New York, USA), washing with distilled water and drying in a microwave oven for 8 min at 350 W.

## Results

### Aerobic and anaerobic batch cultivation

TMB 3415 was cultivated in synthetic medium containing either glucose or xylose as the sole carbon source, under aerobic and anaerobic conditions. Aerobic growth was maintained for approximately 2 cell divisions, after which anaerobiosis was established by switching the sparging gas from air to nitrogen. The fermentation profiles of glucose and xylose growth in this setup are presented in Figure 1. Balanced exponential cell growth was seen for glucose and xylose under both aerobic and anaerobic conditions, allowing for the assumption of pseudo steady-state conditions [24]. Growth rates and metabolic fluxes were calculated for the anaerobic growth phase (Table 2).

**Table 2:** Metabolite production rates and yields during anaerobic growth on glucose and xylose.

	**μ**	***r***_**s**_	***r***_**xol**_	***r***_**g**_	***r***_**a**_	***r***_**e**_	**Y**_**xe**_	**Y**_**xg**_	**Y**_**se**_	**Y**_**sxol**_
**Glucose** **anaerobic**	0.334 ± 0.006	2.6 ± 0.5	0	0.33 ± 0.01	0.01 ± 0.01	1.2 ± 0.2	3.3	0.98	0.43	0
**Xylose** **anaerobic**	0.0246 ± 0.0003	0.285 ± 0.005	0.058 ± 0.003	0.015 ± 0.004	0	0.126 ± 0.005	5.1	0.59	0.44	0.20

Symbols: μ, specific growth rate (h^-1^); r, specific consumption/production rates (g/gDW×h): r_s_, specific substrate consumption rate; r_xol_, specific xylitol production rate; r_g_, specific glycerol production rate; r_a_, specific acetate production rate; r_e_, specific ethanol production rate; Y, yields (g/g): Y_xe_, ethanol yield on biomass; Y_xg_, glycerol yield on biomass; Y_se_, ethanol yield on substrate; Y_sxol_, xylitol yield on substrate.

In glucose medium, growth rates were similar regardless of oxygenation (0.43 h^-1 ^vs. 0.33 h^-1^) (Figure 1A) and comparable to previous reports [35]. During xylose consumption on the other hand, the growth rate decreased from 0.20 h^-1 ^to 0.025 h^-1 ^under anaerobic conditions (Figure 1C). Specific rates of substrate uptake and metabolite production were also different during anaerobic glucose and xylose fermentation (Table 2). The substrate uptake rate and the ethanol production rate were approximately an order of magnitude lower during xylose utilization than during glucose utilization (Table 2). The productivity, 0.13 g/gDW×h, and yield, 0.43 g/g, of ethanol from xylose (Table 2) was however significantly higher compared to several recently investigated xylose-utilizing strains [20,26,36,37]. It is reasonable to assume that anaerobic growth of strain TMB 3415 results from increased ethanol productivity and low xylitol yield, which in turn can be ascribed to the K270R mutation in XR [26]. Compared to strains expressing a xylose isomerase based pathway, the ethanol productivity in TMB 3415 was higher than in non-growing strains [10,11,38], whereas it was similar to a strain growing at μ = 0.03 h^-1 ^[39] and lower than a strain growing at μ = 0.09 h^-1 ^[40].

### Topography of microarray data

The present study aimed at highlighting regulatory differences between anaerobic glucose and xylose growth by xylose-utilizing *S. cerevisiae*. Specifically, differences in growth rate, substrate consumption and ethanol productivity were analyzed on a transcriptional level. Transcriptional characterization have not previously been performed under anaerobic conditions due to the inability of recombinant *S. cerevisiae *strains to grow on xylose in the absence of oxygen [17,20-22]. A few strains expressing *Piromyces *XI are able to grow anaerobically on xylose [39,40], however to the best of our knowledge transcription analysis of these strains has not been reported.

**Figure 2:**
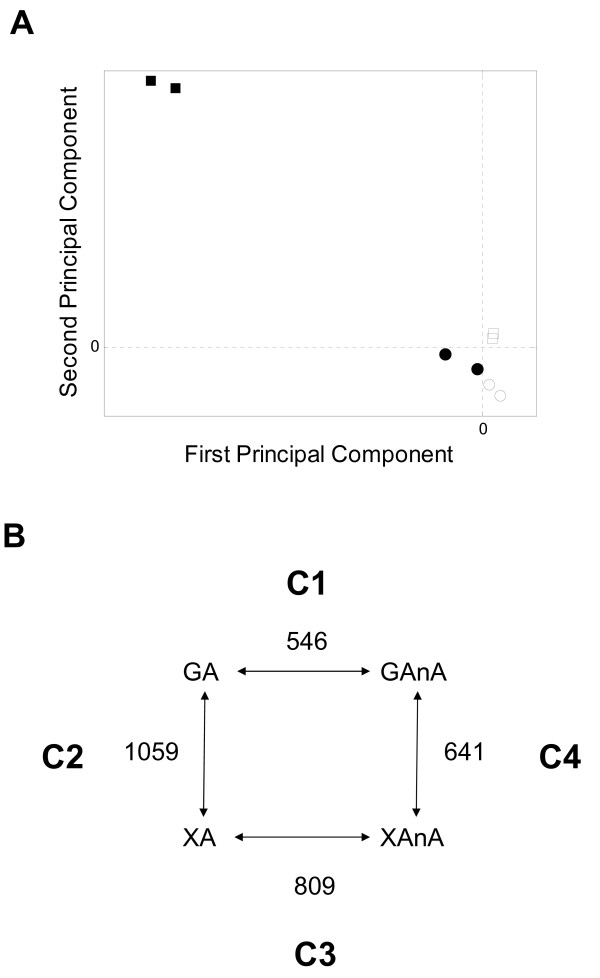
**A PCA projection of individual microarray samples**. The dashed lines separate samples in 4 quadrants depending on the experimental condition. Symbols: Aerobic glucose, "empty circle"; Anaerobic glucose, "filled circle"; Aerobic xylose, "empty square"; Anaerobic xylose, "filled square". **B **The number of differently expressed genes (95% confidence interval) is indicated for pairwise comparisons of experimental conditions. Abreviations: GA, glucose aerobic; GAnA, glucose anaerobic; XA, xylose aerobic; XAnA, xylose anaerobic. C1-C4 designate specific pairwise comparisons, e.g. C1 = glucose aerobic-glucose anaerobic.

The overall structure of the microarray data was examined using Principle Component Analysis (PCA) [41]. PCA has been used to reduce the dimensionality of microarray data and to identify features of experimental conditions that best explain the observed variance in gene expression [42]. The projection of the two principle components, oxygen availability and carbon source, segregated the individual samples in a two dimensional space (Figure 2A). The four conditions, glucose aerobic (GA), xylose aerobic (XA), glucose anaerobic (GAnA) and xylose anaerobic (XAnA), were separated along the axis of the first and second principle component. The first principal component, which was responsible for the most variance in the data set, separated samples according to oxygen availability (aerobic or anaerobic) (Figure 2A). The second principal component separated samples according to carbon source (glucose or xylose). The PCA projection of the transcription data singled out anaerobic xylose growth as the most unique group in the data set (Figure 2A).

Next, the microarray data was organized into four relevant pairwise comparisons: GA vs. GAnA (Comparison 1 = C1); GA vs. XA (Comparison 2 = C2); XA vs. XAnA (Comparison 3 = C3) and GAnA vs. XAnA (Comparison 4 = C4) (Figure 2B). Comparisons C1 and C2 have previously been reported in slightly different experimental setups [17,20,23]. However, comparisons C3 and C4 allowed for the first time analysis of transcription during anaerobic growth on xylose as a sole carbon source. Globally, the transition from aerobic to anaerobic growth changed the expression level of more genes on xylose (C3, 809 genes) than on glucose (C1, 546 genes) (Figure 2B). This difference is presumably due to that aerobic metabolism on xylose is fully respiratory, while it is respiro-fermentative on glucose. Likewise a higher number of genes displayed different expression levels between glucose and xylose utilization under aerobic conditions (C2, 1059 genes) than under anaerobic conditions (C4, 641 genes) (Figure 2B).

**Figure 3:**
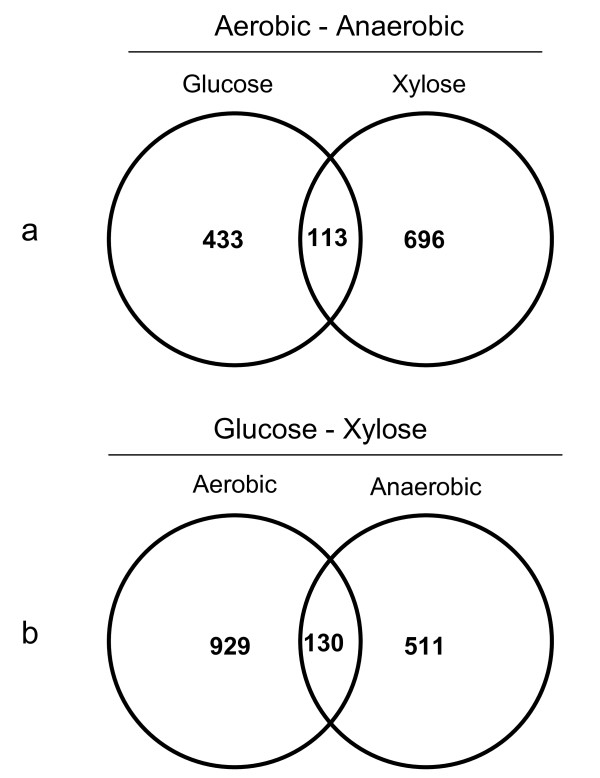
**Venn diagram showing the fraction of genes common for**: (a) transition between aerobic-anaerobic conditions regardless of carbon source; or (b) transition between glucose and xylose regardless of oxygenation level. The total number inside each circle represents the number of genes with significantly changed expression levels in that particular comparison, e.g. 546 genes for the glucose aerobic - glucose anaerobic transition (C1, Figure 2B).

Finally, subsets of genes were isolated according to the following criteria: (i) genes that changed expression levels between aerobic and anaerobic conditions regardless of carbon source (C1&C3, Figure 3A) and (ii) genes that changed expression levels on glucose and xylose regardless of oxygenation level (C2&C4, Figure 3B). Relatively few genes, 113, changed expression on both glucose and xylose during transition from aerobic to anaerobic conditions (Figure 3A). Likewise, only 130 genes changed expression level on xylose compared to glucose irrespective of oxygenation level (Figure 3B).

### Gene ontology (GO) terms

Within a group of genes, up- and down-regulated pathways and processes can be identified by searching for over-represented gene ontology (GO) terms . Each annotated gene in the *S. cerevisiae *genome is associated with one or several GO terms that describe the corresponding biological process, e.g. amino acid synthesis. Significantly enriched gene ontology terms (p < 0.01) were identified within the previously described groups (C1, C2, C3, C4, C1&C3 and C2&C4) (Table 3). If more than one GO term in the same "family" were identified, only the most significant term was listed.

**Table 3:** Gene ontology (GO) terms.

**Comparison**	**Regulation**	**GO ID**	**Term**
Glucose Aerobic-Anaerobic (C1)	Up	ND	ND
	
	Down	GO:0055114	oxidation reduction
		
		GO:0015980	energy derivation by oxidation of organic compounds
		
		GO:0006119	oxidative phosphorylation

Xylose Aerobic-Anaerobic (C3)	Up	GO:0006066	cellular alcohol metabolic process
		
		GO:0019320	hexose catabolic process
		
		GO:0007047	cell wall organization
	
	Down	GO:0042254	ribosome biogenesis
		
		GO:0008652	amino acid biosynthetic process
		
		GO:0016072	rRNA metabolic process

C1&C3	Up	ND	ND
	
	Down	GO:0043102	amino acid salvage
		
		GO:0045333	cellular respiration

Aerobic Glucose - Xylose (C2)	Up	GO:0007005	mitochondrion organization
		
		GO:0045333	cellular respiration
		
		GO:0022900	electron transport chain
	
	Down	GO:0032268	regulation of cellular protein metabolic process
		
		GO:0006066	cellular alcohol metabolic process

Anaerobic Glucose - Xylose (C4)	Up	GO:0015980	energy derivation by oxidation of organic compounds
		
		GO:0019318	hexose metabolic process
	
	Down	GO:0042254	ribosome biogenesis
		
		GO:0008652	amino acid biosynthetic process
		
		GO:0016072	rRNA metabolic process

C2&C4	Up	GO:0006119	oxidative phosphorylation
	
	Down	ND	ND

Up- and down-regulated gene ontology (GO) terms identified within comparisons C1-C4 (Figure 2B). If no significantly over-represented GO terms were found, this was indicated by "ND" for "not detected".

Comparing aerobic and anaerobic metabolism on glucose (C1, Figure 2B), unilateral down-regulation of respiratory processes was identified (Table 3). Down-regulation of respiratory genes in response to anaerobiosis has previously been reported for glucose grown cells in batch culture [17] and C- and N-limited chemostat culture [42,43]. In contrast, respiratory pathways were not down-regulated between aerobic and anaerobic xylose metabolism (C3, Figure 2B), whereas processes related to protein synthesis were significantly repressed (Table 3). Down-regulation of ribosome biogenesis and amino acid synthesis [42,44] reflects the ten fold reduction of growth rate between aerobic and anaerobic conditions on xylose (Figure 1B). In addition, the GO terms "alcohol metabolism" and "hexose catabolism" were up-regulated under anaerobic conditions on xylose (Table 3). In the group of genes that changed expression in response to anaerobiosis irrespective of carbon source (C1&C3, Figure 3A), repression of cellular respiration was identified (Table 3).

Aerobic glucose metabolism was respiro-fermentative, while aerobic xylose metabolism was completely respiratory with absent ethanol production (Figure 1). On a transcription level, this difference was visible in higher expression of several respiration related GO terms on xylose compared to glucose during aerobic conditions (C2, Table 3) [17,20]. During anaerobic conditions, protein synthesis was down-regulated on xylose compared to glucose, while expression of respiratory processes and the GO term "hexose metabolism" was up-regulated (C4, Table 3). The lower expression of amino acid biosynthesis [42,44] is related to the lower growth rate on xylose compared to glucose under anaerobic conditions. Oxidative phosphorylation was up-regulated on xylose in the group of genes that were differently expressed on glucose and xylose under both aerobic and anaerobic conditions (C4&C2, Table 3).

Respiration was not repressed between aerobic and anaerobic growth on xylose (C3, Table 3), and higher expression of respiratory processes was observed during both aerobic and anaerobic conditions compared to glucose (C4&C2, Table3). Certain processes in cellular respiration were however down-regulated both on glucose and xylose in response to anaerobiosis (C1&C3, Table 3). This indicates that although oxidative metabolism was not unilaterally down-regulated under anaerobic conditions, some processes were still repressed.

### Regulation of the central carbon metabolism

**Figure 4:**
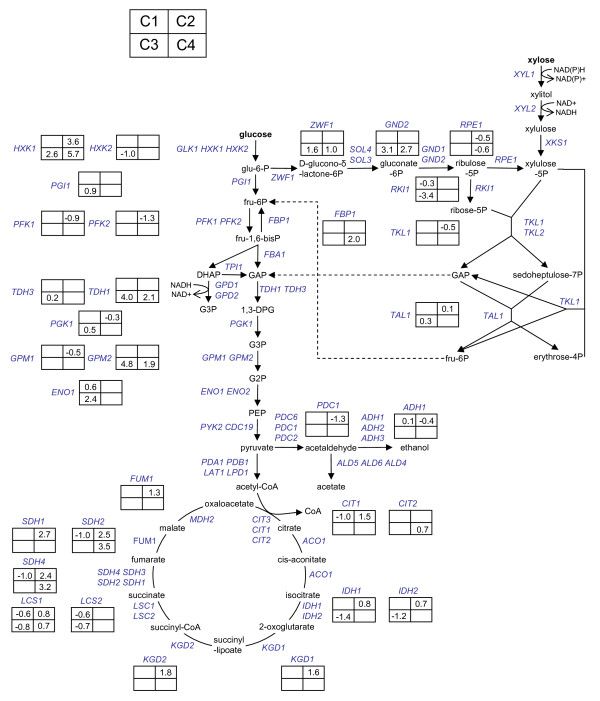
**Regulation of central metabolism under aerobic/anaerobic growth on glucose/xylose**. The fold change (log2) of expression is presented for the C1, C2, C3 and C4 comparisons. Cofactor utilization is only depicted for relevant reactions. Standard three letter code is used for all genes names .

Expression of the GO term "hexose metabolism" increased specifically under anaerobiosis on xylose, both in comparison to anaerobic glucose utilization (C4) and to xylose under aerobic conditions (C3) (Table 3). This observation was dependent on anaerobic xylose growth and has thus not previously been reported. The GO term "hexose metabolism" encompasses several aspects of the central carbon metabolism (glycolysis, pentose phosphate pathway, gluconeogenesis etc.), which is responsible for the cell's energy metabolism. As such, regulation of central carbon metabolism is linked to growth rate, protein synthesis and ethanol production rate [44,45]. Several reaction steps in the central carbon metabolism are catalyzed by more than one isozyme, which enable the cell to regulate the direction/flux of the pathway in response to the energetic state of the cell. Therefore, expression of genes in the central carbon metabolism was investigated in greater detail to pinpoint differences between glucose and xylose anaerobic growth (Figure 4).

Increased reversed flux in glycolysis during anaerobic xylose utilization was indicated by expression of several isozymes specific for gluconeogenesis. The hexokinase gene *HXK1 *was expressed higher on xylose than on glucose irrespective of aeration (Figure 4). Previously, high *HXK1 *expression has been reported under aerobic xylose growth [17,20] and during metabolism of non-fermentable carbon sources [46]. Further down the pathway, the exclusively gluconeogenetic enzyme fructose-1,6-bisphosphatase *FBP1 *was up-regulated specifically during anaerobic xylose utilization (Figure 4). Expression of glyceraldehyde-3-phosphate dehydrogenase isozyme *TDH1 *is linked to stationary growth and gluconeogenesis [47] and was similarly up-regulated on xylose during anaerobic conditions (Figure 4). Also the minor isoform of phosphoglycerate mutase *GPM2 *was up-regulated on xylose during anaerobic conditions (Figure 4) but the function of this enzyme is largely unknown [48].

While reversed flux in glycolysis was indicated during anaerobic xylose growth, increased activity of the oxidative pentose phosphate pathway was observed through expression of 6-phosphogluconate dehydrogenase(*GND2*) and glucose-6-phosphate dehydrogenase (*ZWF1*) (Figure 4). Both *GND2 *and *ZWF1 *catalyze the reduction of NADP^+ ^and were specifically up-regulated under anaerobic conditions on xylose (Figure 4). During anaerobic conditions, NADP^+ ^reduction in the oxidative pentose phosphate pathway controls the rate of xylose utilization as a consequence of the cofactor imbalance in the XR-XDH reaction [49]. Increased flux in the oxidative pentose phosphate pathway (PPP) must however be supported by proportionally increased reversed flux in glycolysis, which indeed was seen in the current study.

## Discussion

In the present study, metabolic fluxes and genome-wide transcription analysis were investigated in recombinant *S. cerevisiae *under strictly anaerobic conditions with xylose as a sole carbon source. The currently employed strain is the first strain utilizing an oxidoreductive xylose-assimilating pathway capable of sustained anaerobic xylose growth. Anaerobic growth on xylose has previously been described for strains expressing an isomerase based pathway [39,40], however to the best of our knowledge, transcription analysis have not been reported for these strains. In previous transcription studies, steady state condition on xylose has been achieved by addition of oxygen or glucose to support growth [17,20-22]. The difference between aerobic utilization of glucose and xylose is thus well described [17,20], whereas the current study represents the first complete characterization of anaerobic xylose growth.

Calculated metabolic fluxes showed that substrate uptake rate (2.6 g/gDW×h vs. 0.29 g/gDW×h) and ethanol productivity (1.2 g/gDW×h vs. 0.13 g/gDW×h) on glucose and xylose were proportional to the growth rates (0.33 h^-1 ^vs. 0.025 h^-1^) (Table 2). Transcription data likewise verified that the low anaerobic growth rate on xylose correlated directly to reduced expression of genes for amino acid synthesis and protein synthesis (Table 3), which has previously been shown for glucose grown cultures [42]. The ethanol yield from consumed substrate (0.43 g/g) on the other hand was identical in anaerobic glucose and xylose fermentation. On xylose, 20% substrate was lost as xylitol, however on glucose this was approximately balanced by almost two times higher glycerol yield compared to xylose (Table 2). The lower glycerol yield during xylose utilization is most likely due to that xylitol acts as a redox sink for anabolic reactions analogously to glycerol [50]. Supporting this argument, it has previously been seen that addition of an external redox acceptor reduced both glycerol and xylitol formation in cultivation of xylose utilizing *S. cerevisiae *[51,52].

Transcription analysis showed that oxidative phosphorylation was de-repressed on xylose compared to glucose under aerobic conditions, which correlates with the absence of respiro-fermentative metabolism on xylose (Table 3) [17,18,20]. During anaerobic conditions however, most respiratory genes continued to be highly expressed on xylose while they were unilaterally repressed on glucose (Table 3). The maintenance of unrepressed oxidative metabolism during anaerobic xylose growth can not be completely explained by lack of catabolite repression [53] since exclusively oxygen dependent repression was seen on glucose. It is however possible that on xylose, expression of "oxidative metabolism" was partly maintained as compensatory response to the cofactor imbalance during anaerobic conditions. Altered redox metabolism and up-regulation of genes for NADPH formation and NADH oxidation was previously seen during oxygen-limited xylose growth [17] and in an evolutionary engineered xylose-utilizing strain [21]. In the current study, the NAD+/NADH dehydrogenase shuttle (*NDI1*, *NDE1 *and *NDE2*) and NADP+ linked glutamate dehydrogenase (*GDH3*) were down-regulated on glucose under anaerobic conditions but up-regulated on xylose (data not shown).

During xylose utilization, up-regulation of gluconeogenesis and the oxidative pentose phosphate pathway coincided with anaerobiosis (Figure 4). On xylose, increased flux in the oxidative PPP is explained by need for NADP+ reduction in anerobic co-factor recycling [49]. Increased reversed flux in upper glycolysis follows consequently from mass balance at the glucose-6-phosphate node (Figure 4). As such, it has previously been seen that high flux in the oxidative PPP, and consequently gluconeogenesis, lowered net glycolytic flux and ethanol productivity [54]. The connection between cofactor recycling, gene expression and metabolic flux offers an explanation to the low ethanol productivity on xylose compared to glucose, despite similar ethanol yields (Table 2). Similarly it is possible that reduced back-flow in glycolysis is the primary reason for the increased ethanol productivity and anaerobic growth rate in the mutant XR (K270R) utilized in the current study [26]. Further improvement of cofactor imbalance in the initial two steps of xylose utilization by protein engineering is expected to improve performance of *S. cerevisiae *strains utilizing an oxido-reductive xylose assimilating pathway.

## Conclusion

The present work describes the metabolic and transcriptional characterization of the xylose utilizing strain TMB 3415 under aerobic and anaerobic conditions. Under anaerobic conditions, metabolism and growth rate on xylose were proportionally reduced compared to glucose, which was reflected in terms of repressed protein synthesis. In addition, cofactor imbalance during anaerobic xylose growth may have caused up-regulation of oxidoreductive metabolism, pentose phosphate pathway and gluconeogenesis. To further investigate regulation of xylose metabolism in anaerobic conditions, strains using oxidoreductive and isomerase based xylose assimilating pathways should be compared at the transcriptional level. The regulatory effect of cofactor imbalance could thus be assayed.

## Abbreviations

1,3-DPG: 1,3-bisphosphoglycerate; CoA: coenzyme A; DHAP: dihydroxyacetone-phosphate; G3P: 3-phosphoglycerate; G2P: 2-phosphoglycerate; GAP: glyceraldehyde 3-phosphate; fru-6P: fructose 6-phosphate; fru-1,6-bisP: fructose 1,6-bisphosphate; glu-6P: glucose 6-phosphate; HPLC: high performance liquid chromatography; NADH: nicotinamide adenine dinucleotide; NADPH: nicotinamide adenine dinucleotide phosphate; OD: optical density; - P: phosphate; PEP: phosphoenolpyruvate; PPP: pentose phosphate pathway; XDH: xylitol dehydrogenase; XI: xylose isomerase; XK: xylulokinase; XR: xylose reductase; YNB: yeast nitrogen base.

## Competing interests

The authors declare that they have no competing interests.

## Authors' contributions

DR participated in the design of the study, performed the experimental work and wrote the manuscript. BHH participated in the design of the study and commented on the manuscript. MB participated in the design of the study, performed the experimental work and commented on the manuscript. All the authors have read and approved the final manuscript.
